# Micellar Carriers of Active Substances Based on Amphiphilic PEG/PDMS Heterograft Copolymers: Synthesis and Biological Evaluation of Safe Use on Skin

**DOI:** 10.3390/ijms22031202

**Published:** 2021-01-26

**Authors:** Justyna Odrobińska, Magdalena Skonieczna, Dorota Neugebauer

**Affiliations:** 1 Department of Physical Chemistry and Technology of Polymers, Faculty of Chemistry, Silesian University of Technology, 44-100 Gliwice, Poland; justyna.odrobinska@polsl.pl; 2 Department of Systems Biology and Engineering, Silesian University of Technology, Akademicka 16, 44-100 Gliwice, Poland; Magdalena.Skonieczna@polsl.pl; 3 Biotechnology Centre, Silesian University of Technology, Krzywoustego 8, 44-100 Gliwice, Poland

**Keywords:** polydimethylsiloxane, heterografted copolymers, micellar carriers, Franz diffusion cells, cytotoxicity

## Abstract

Amphiphilic copolymers containing polydimethylsiloxane (PDMS) and polyethylene glycol methyl ether (MPEG) were obtained via an azide-alkyne cycloaddition reaction between alkyne-functionalized copolymer of MPEG methacrylate and azide-functionalized PDMS. “Click” reactions were carried out with an efficiency of 33–47% increasing grafting degrees. The grafted copolymers were able to carry out the micellization and encapsulation of active substances, such as vitamin C (VitC), ferulic acid (FA) and arginine (ARG) with drug loading content (DLC) in the range of 2–68% (VitC), and 51–89% (FA or ARG). In vitro release studies (phosphate buffer saline, PBS; pH = 7.4 or 5.5) demonstrated that the maximum release of active substances was mainly after 1–2 h. The permeability of released active substances through membrane mimicking skin evaluated by transdermal tests in Franz diffusion cells indicated slight diffusion into the solution (2–16%) and their remaining in the membrane. Studies on the selected carrier with FA showed no negative effect on cell viability, proliferation capacity or senescence, as well as cell apoptosis/necrosis differences or cell cycle interruption in comparison with control cells. These results indicated that the presented micellar systems are good candidates for carriers of cosmetic substances according to physicochemical characterization and biological studies.

## 1. Introduction

Polymeric carriers of active substances are prepared primarily via controlled polymerization methods, including atom transfer radical polymerization (ATRP), reversible addition-fragmentation chain transfer radical polymerization or ring-opening polymerization [1,2]. These methods allow the formation of well-defined polymers with different chemical characteristics and required architecture. However, polymeric systems have been studied mainly for the delivery of anti-cancer drugs [3,4]. In the field of cosmetology, polymeric carriers are commonly based on biopolymers, such as polysaccharides, poly(lactic acid) and chitosan [5,6]. One of the most important aspects in all biomedical applications is the selection of appropriate non-toxic, biocompatible polymers, which can form micelles or conjugates—common types of carriers.

Hydroxy- and methoxy-functionalized poly(ethylene glycol) methacrylates (PEGMA and MPEGMA, respectively) are the most studied to prepare block and graft copolymers [1]. They are beneficial to attain hydrogels [7,8], nanogels [9,10], nanocontainers [11] and nanoparticles [12]. Moreover, PEGMA and MPEGMA have been applied to the preparation of polymers for self-assembly [13,14], drug delivery [15,16,17,18,19] and protein conjugation [20,21]. Due to MPEGMA’s low cytotoxicity, it can be successfully used in materials for medical and cosmetic applications [22] as well as to prepare biomaterials, for example, in combination with keratin [23].

A wide range of biomedical and healthcare applications has been reported for polydimethylsiloxane (PDMS) due to its good blood and tissue compatibility, as well as its chemical, thermal and oxidative stability and antiadhesive properties [24,25]. Moreover, PDMS has been characterized to possess low toxicity, good ability of film forming and flexible properties owing to the presence of Si-O-Si bonds [26]. The latest literature reports indicate the possibility of using PDMS in new engineering solutions, such as high-performance CO_2_ separation membranes obtained by incorporating micelles based on copolymer of PDMS/poly(ethylene glycol)-behenyl ether methacrylate into the interlamellar regions of poly(ethylene oxide) crystallites [27], magnetic PDMS membranes to mediate cell behavior [28], high flexible pressure sensors obtained by combining conical microstructure PDMS with bilayer graphene [29], as well as the fabrication of PDMS microchannels for biomedical application (varicose veins implantation) [30]. PDMS-based systems are often used as micellar carriers of anti-cancer substances (e.g., doxorubicin) [31], nanoparticles (e.g., with cathepsin B, lysosomal cysteine proteases that associate with premalignant lesions and invasive stages of cancer) [32], and PDMS-modified silica xerogels with Ag nanoparticles (progesterone delivery) [33]. Furthermore, PDMS-based coatings and silica-PDMS composites have also been tested in drug delivery systems and biomedicine [34,35,36]. Recent research validates that the toxicity of PDMS coatings is dependent on the concentration [36] and that the increase in the molecular weight of PDMS can improve the cell survival rate [37]. Additionally, special anti-fouling properties of PDMS products have been achieved for elastomers with polyurethane urea [38], as well as for PEGMA brushes obtained via the surface-initiated ATRP technique on roller-casted multiple PDMS layers [39]. Cosmetic products containing PDMS create a film on the surface of the skin, which protects against microbes and moisture loss without interfering with the skin’s physiological functions, while improving its appearance and condition [40].

**Figure 1:**
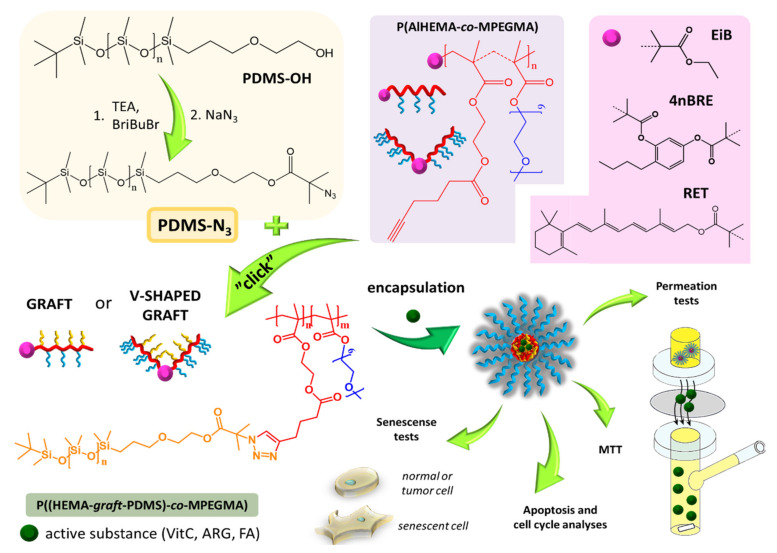
Synthesis of poly(ethylene glycol) (PEG) graft copolymers with polydimethylsiloxane (PDMS) as additional side chains via “click” reaction and their use in encapsulation and release of bioactive substances.

In our previous work, we described the strategy of grafting poly(ethylene glycol) (PEG) side chains onto the copolymer of methyl methacrylate (MMA) and alkyne functionalized 2-hydroxyethyl methacrylate (AlHEMA) (P(AlHEMA-*co*-MMA) [41,42]. The preparation of PEG/poly(ε-caprolactone) heterograft copolymers was also examined [43]. The aim of our current work was to prepare other types of amphiphilic copolymers containing PDMS as extra side chains (PEG/PDMS), which could self-assemble into micellar structures (Figure 1). The copolymers of AlHEMA and MPEGMA were obtained using various ATRP initiators (ethyl 2-bromoisobutyryl (EiBBr), bromoester functionalized retinol (RETBr) and 4-*n*-butylresorcinol (4nBREBr_2_)). The technique of “grafting through” MPEGMA macromonomers provided the formation of PEG graft or V-shaped PEG graft copolymers depending on the amount of bromoester groups in the initiator. Bearing in mind the high flexibility of PDMS chains, they were employed to modify PEG graft copolymers to provide the heterografted systems (PEG/PDMS). The combination of PEG and PDMS was achieved by the “grafting onto” technique using the “click” reaction between azide functionalized PDMS and alkyne contained on the main chain of polymethacrylate (P(AlHEMA-*co*-MPEGMA)). The ability of self-assembly allowed the encapsulation of selected active substances (vitamin C, VitC; ferulic acid, FA; arginine, ARG). The release of the loaded substance was tested under conditions similar to those on human skin (phosphate buffer saline, PBS; pH = 7.4 and pH = 5.5 at 37 °C). The loading efficiencies and kinetic profiles were indicated to evaluate the potential of the described systems for the delivery of active substances in cosmetology. The micellar systems characterized by the best parameters in terms of their use as carriers of active substances in cosmetic products were selected to conduct penetration tests through a membrane imitating artificial skin. Next, one sample beneficial for application in cosmetic products was evaluated by cytotoxicity tests on healthy and melanoma human skin cells, cell cycle and cell senescence tests as well as research into the type of cell death (apoptosis vs. necrosis).

## 2. Results

Amphiphilic PEG graft copolymers (P(AlHEMA-*co*-MPEGMA)) were obtained by controlled ATRP of MPEGMA macromonomers using different initiators (EBiBr, RETBr, and 4nBREBr_2_). The control of alkyne groups’ content as well as the hydrophilicity of copolymers was assured by initial proportions of monomers (AlHEMA/MPEGMA: 25/75, 50/50). The structure of copolymers (I-V) was confirmed by ^1^H NMR spectroscopy, where the proton resonances corresponding to methyl groups in the copolymer main chain were observed at B_p_: 0.6–1.5 ppm, AlHEMA -COOCH_2_CH_2_OCO- groups at E_p_: 3.86–4.24 ppm, and MPEGMA -OCH_2_CH_2_O- groups in side chains at C_p_: 3.51 ppm (Figure S1). The monomers’ conversion, molecular weight (M_n_), polymerization degree (DP_n_) and grafting degree (DG_PEG_) were calculated using gas chromatography (GC) analysis.

**Figure 2:**
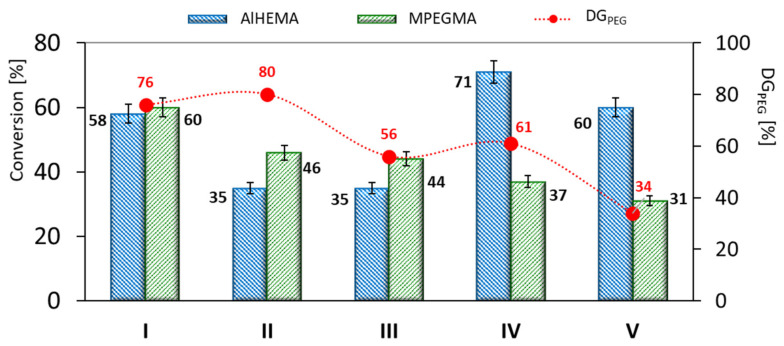
Conversion of AlHEMA and MPEGMA comonomers, and grafting degree (DG_PEG_ = (DP_MPEGMA_/DP_n_) * 100%) in the resulted copolymers.

The conducted polymerization reactions displayed AlHEMA conversions in the range of 35–71% and 26–60% for MPEGMA, which resulted in DP_n_ above 150 (Table 1). The grafted copolymers (I-III) obtained using monofunctional initiators (EiBBr or RETBr) presented a statistical distribution of PEG grafts due to comparable conversions of AlHEMA vs. MPEGMA (Figure 2). In the case of V-shaped graft copolymers (IV-V) obtained with bifunctional 4nBREBr_2_, the lower conversion of the macromonomer (MPEGMA) is responsible for the gradient structure with primarily AlHEMA units. The use of so-called “bio”initiators (RETBr, 4nBREBr_2_), compared with standard EiBBr, caused a prolonged reaction time to achieve DP_n_ ~180 (3–24 h vs. 2.5 h). The systems with equimolar initial proportions of AlHEMA/MPEGMA (III, V) resulted in obtaining copolymers with lower grafting density (DG_PEG_ = 34–56%) in comparison with I, II and IV (Figure 2).

The majority of the copolymers showed relatively low dispersity indices (1.28–1.44, Table 1), which were illustrated by monomodal and symmetrical signals of gel permeation chromatography (GPC) traces (Figure S2). In the case of copolymers with an equimolar initial proportion of AlHEMA and MPEGMA (III, V), a broadening of the signal corresponding to higher dispersity (1.76–1.88) led us to conclude the occurrence of a larger content of side reactions due to the influence of the AlHEMA steric hindrance caused by rigid triple bonds. The discrepancy between the M_n_ values calculated from conversion analysis by GC and those determined by GPC analysis could be explained by the grafted topology of the copolymers, which had a lower hydrodynamic volume in solution compared to linear polymer standards used in GPC calibration.

Modification of P(AlHEMA-*co*-MPEGMA)s by the attachment of flexible chains of PDMS via Cu(I) catalyzed 1,3-dipolar azide-alkyne cycloaddition (CuAAC) was expected to design heterografted copolymers with more specific properties (Figure 1). Therefore, an important aspect of this work was the preparation of properly functionalized PDMS. For this purpose, a commercially available hydroxy-terminated PDMS was subjected to the esterification reaction introducing a bromoester group (PDMS-Br), which was then converted via the substitution reaction to an azide group (PDMS-N_3_). The formation of azide-functionalized PDMS-N_3_ was confirmed by ^1^H NMR analysis, which showed the appearance of resonance from methyl protons of the isobutyrate group (H_J_: 2.10 ppm, Figure S3b), and then its removal due to the exchange of the bromine to an azide group (H_J_: 2.02 ppm, Figure S3c). The structure of PDMS-N_3_ was additionally confirmed by ^13^C NMR analysis, where the signal of methyl carbons from the isobutyrate group was observed at 22 ppm (C13) (Figure S4). The modified polymers were characterized by low dispersity below 1.15 and narrow symmetrical GPC traces (Table 2, Figure S5).

**Figure 3:**
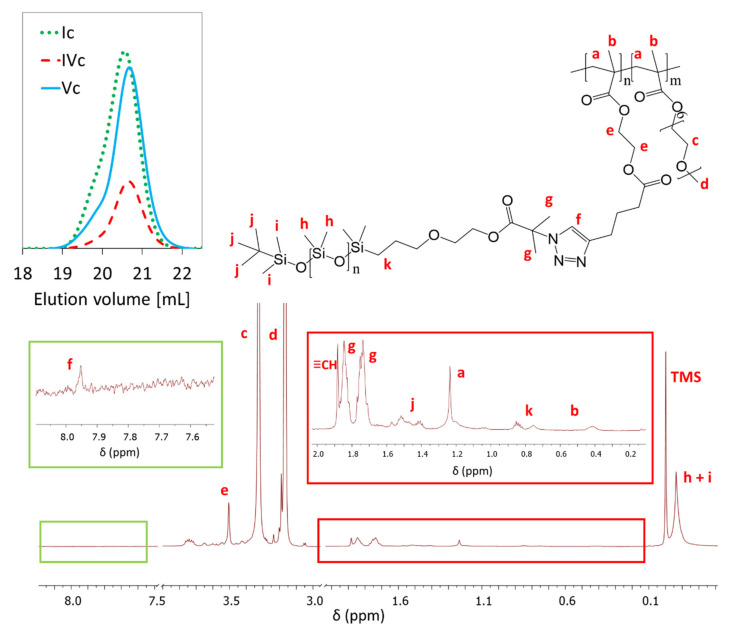
^1^H NMR of P((HEMA-*graft*-PDMS)-*co*-MPEGMA) IIc and GPC traces of representative “click” copolymers.

Functionalized PDMS-N_3_ was grafted onto previously described P(AlHEMA-*co*-MPEGMA)s using the “click” reaction catalyzed by CuBr/PMDTA, which leads to the formation of 1,4-substituted triazole rings. As a result, heterografted copolymers P((HEMA-*graft*-PDMS)-*co*-MPEGMA) with amphiphilic properties were achieved. The presence of the triazole was confirmed by ^1^H NMR analysis of the “click” reaction product by the peak at 7.95 ppm (H_f_) (Figure 3). Additional confirmation was observed by the signals at 0.10 ppm (H_h+j_), 1.74 and 1.85 ppm (H_g_) and 0.75 and 0.85 ppm (H_k_) corresponding to the PDMS side chain and the other intensive signals at 3.51 ppm (H_e_), 1.28 ppm (H_a_) and 0.42 ppm (H_b_) corresponding to protons in the polymer main chain.

The effectiveness of the “click” reaction (E_click,_ Table 3) was calculated using the triazole ring’s proton signal at 7.95 ppm (H_f_) and signals from unreacted alkyne groups at 1.88 ppm (H_≡CH_). The PDMS “click” was efficient in 33–47%. Comparing copolymers with different architecture i.e., grafted Ic-IIIc vs. V-shape grafted IVc, Vc, a correlation was evident, showing the highest value of E_click_ for the latter ones (33–40% vs. 43–47%, respectively). This regularity may be due to the statistical distribution of AlHEMA units in the case of graft copolymers (Ic-IIIc) and the gradient distribution of V-shaped graft copolymers (IVc-Vc). Therefore, the gradual changing frequency of AlHEMA units in the chain was beneficial for the “click” reaction due to easier access of azide-functionalized PDMS to alkyne moieties.

**Figure 4:**
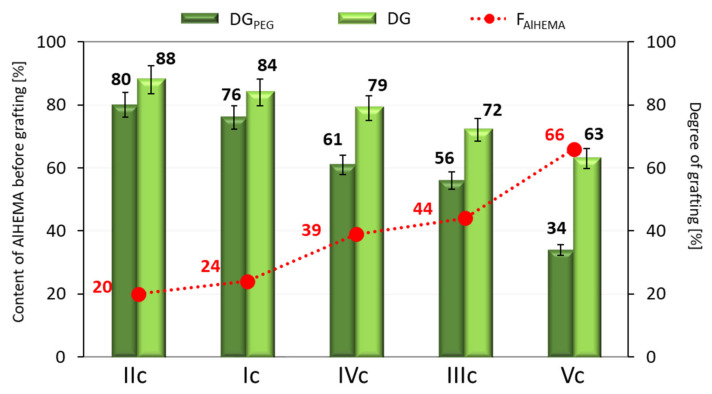
Effect of F_AlHEMA_ on DG of copolymers containing PEG vs. PEG/PDMS side chains.

It was also observed that the degree of grafting (DG) after the “click” reaction decreased with increasing AlHEMA content in the copolymer before the reaction, thus it was concluded that the “click” of P(AlHEMA-*co*-MPEGMA) copolymers was favored by a smaller share of alkyne groups (Figure 4). In addition, the highest grafting degree of PDMS (>20%) was associated with the lowest DG_PEG_, resulting in the lowest total DG of the final copolymer (~63%). GPC analysis indicated the formation of monomodal molecular weight distribution heterografted copolymers with lower or comparable dispersity indices to that of P(AlHEMA-*co*-MPEGMA)s used for “grafting onto” by the “click” strategy (Table 3, Figure 3).

The self-assembling behaviors of the graft copolymers were investigated by detecting the critical micelle concentration (CMC). Copolymers’ CMC values were determined using the emission spectra of pyrene in fluorometric measurements to draw the plot of I_336_/I_332_ vs. logarithm of copolymer solution concentration (logC) (Figure S6). The results showed that the CMC values of graft and V-shaped graft copolymers decreased with increasing PDMS grafting as hydrophobic side chains (DG_PDMS_), which confirmed that micelles were formed more easily by more hydrophobic systems due to lower F_hphil._ associated with lower CMC (Table 3).

**Figure 5:**
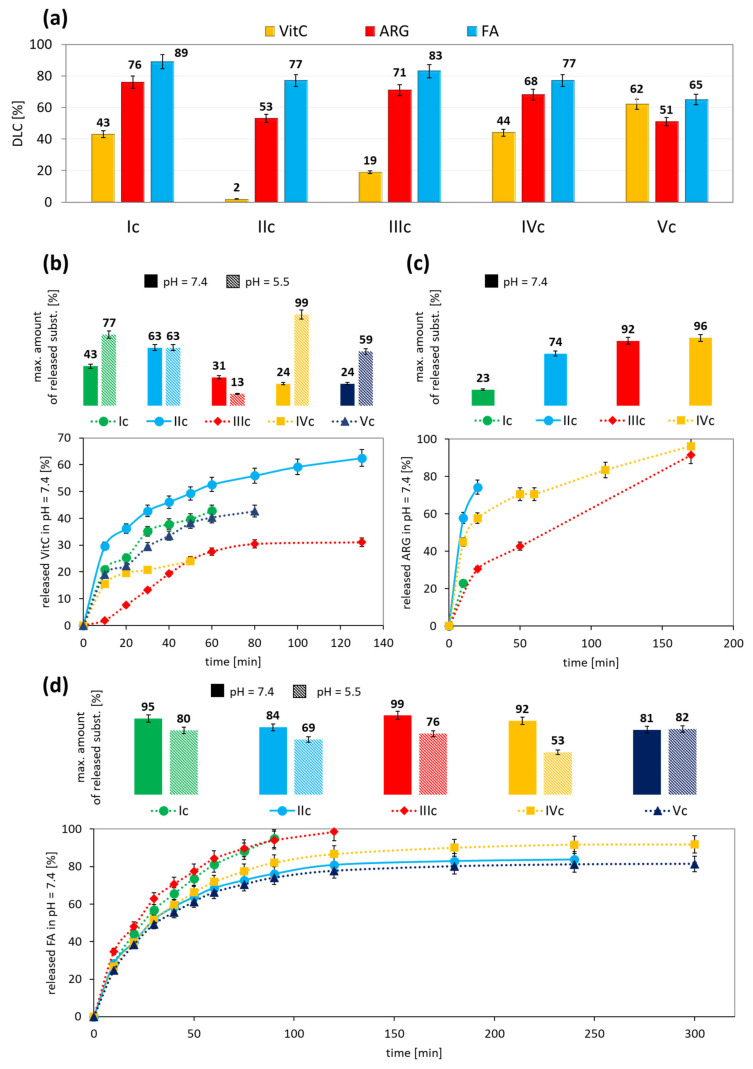
Drug loading content (DLC) of micelles based on PEG/PDMS heterografted polymers (**a**) and kinetic profiles of vitamin C (VitC) (**b**), arginine (ARG) (**c**), and ferulic acid (FA) (**d**) released from polymer micelles in pH = 7.4, and comparison of maximum amount of released substances in pH = 7.4 vs. 5.5.

The ability of the copolymers to self-assemble was advantageous to micelle formation with loaded bioactive substances. VitC, ARG, and FA were selected due to their beneficial properties to the skin, i.e., reduction in discoloration, stimulation of collagen synthesis, and antioxidant properties. The encapsulation efficiency was characterized by the drug loading content (DLC) using UV-Vis spectroscopy (Figure 5a). It was observed that the copolymer topology, i.e., grafted (Ic-IIIc) vs. V-shape grafted (IVc-Vc), did not affect the encapsulation efficiency of ARG and FA, except for VitC, which was more efficiently encapsulated by micelles created by RET- or EiB-based series (2–43% vs. 44–62%). The highest DLC was achieved by micelles with FA (65–89%), which showed the lowest hydrophilicity, and in the series of FA systems it was encapsulated with the lowest DLC in micelles formed by a copolymer with the highest degree of grafted PDMS (28%). The hydrophilicity of the encapsulated substances (VitC > ARG > FA), and thus their solubility in water, causes their DLC for the studied systems to increase in the following order: VitC < ARG < FA (Figure 5a).

The apparent hydrodynamic diameters (D_h_) of the self-assembling particles were determined by DLS in aqueous solution (Table 4). In most cases, the analysis confirmed the formation of homogeneous particles and monomodal signals with the exception of micelles created mainly by V-shaped graft copolymers with loaded VitC (IIc, IIIc, Vc) and FA (IIIc, Vc), where small amounts of unimers or aggregates (<10 VitC and FA%) were observed (Figure S7). The majority of micelles displayed a diameter in the range of 200–300 nm (Table 4, Table S1). Copolymers Ic and IIc, with the highest DG (84–88%) but lowest PDMS grafting degree (8%), resulted in obtaining micelles with the largest D_h_ in the case of VitC and FA encapsulation (>450 nm). Moreover, for the latter FA-based systems it also corresponded to the highest DLC values (>80%). An inverse relationship was observed for micelles with ARG, where a higher DLC was associated with smaller micellar nanoparticles, probably due to ARG’s strong hydrophilic properties.

The release experiments were carried out in PBS at pH = 7.4 and 5.5. The lower pH is closer to that actually present on facial skin, for which the described micellar systems are dedicated. For VitC loaded micelles, the release was completed mainly in the first hour of the experiment regardless of pH, and the maximum amount of released VitC was primarily higher at pH = 5.5 (Figure 5b, Figure S8a, Table S2). The acidic properties of VitC in neutral form favored its release under an acidic environment. Considering the hydrophobic/hydrophilic balance, it was observed that micelles formed by more hydrophilic copolymers (F_hphil._) released a greater amount of hydrophilic VitC (IIc vs. IIIc, IVc vs. Vc). The release experiments carried out for micelles with ARG showed ARG release only at pH = 7.4 (Figure 5c, Table S2). In a buffer with lower pH, a signal was not observed in UV-Vis analysis, thus ARG released at this pH was probably not possible due to ARG’s strong alkaline properties. The maximum amount of ARG was released at pH = 7.4 after only 10 min for systems with the lowest DG_PDMS_ (Ic-IIc), and up to 3 h for the systems with a higher PDMS grafting degree (IIIc-IVc). The micelles with encapsulated FA released the maximum amount of substance within 2 h, but under acidic conditions FA was released with lower efficiency (Figure 5d, Figure S8b, Table S2). Additionally, regardless of the topology and composition of the micelle-forming copolymers, FA was released from the micelles at very high efficiency (over 80% in pH = 7.4, and 53–82% in pH = 5.5). Despite the acidic properties of FA, the lower hydrophilicity compared to VitC may cause FA’s release in a smaller amount at a slightly acidic pH.

**Figure 6:**
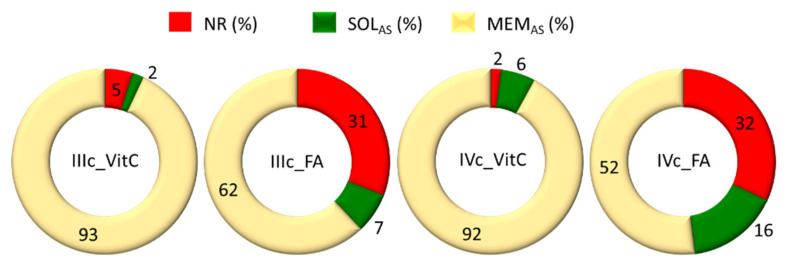
Graph of the amount of non-released substance (NR), amount of released substance passing through the membrane into the solution (SOL_AS_) and amount of released substance remaining in the membrane (MEM_AS_).

Micelles with VitC and FA, formed by graft copolymer IIIc and V-shaped graft copolymer IVc, were selected for further research in Franz diffusion cells. These carriers were characterized by high DLC, where a large amount of released substance occurs during the release tests by dialysis with a cellulose membrane bag, and D_h_ ~200 nm. The permeability studies through the membrane mimicking skin showed that the active substance was released from the carrier in a high degree (^FC^Rmax: 68–98%), which was in contrast to the dialysis method, in which VitC was released in a greater amount compared to FA (95 vs. 69%, 98 vs. 68%) (Table 5). This dependence may be due to the fact that practically all the released VitC was retained in a membrane imitating artificial skin (it was not diffused into the solution) (Figure 6). Thus, during the dialysis studies it was not possible to penetrate the cellulose membrane, VitC was released into the solution in a smaller amount, and the rest remained in the solution in the cellulose membrane bag. Using Franz cells, FA was released in slightly smaller amounts, but the majority of FA diffused through the artificial skin into the solution. The diffusion rate through the membrane was considerably greater for FA (23–33 vs. 0.8–1.0) and higher when the proportion of hydrophilic fraction (HLB) was greater in the copolymer system (Table 5).

**Figure 7:**
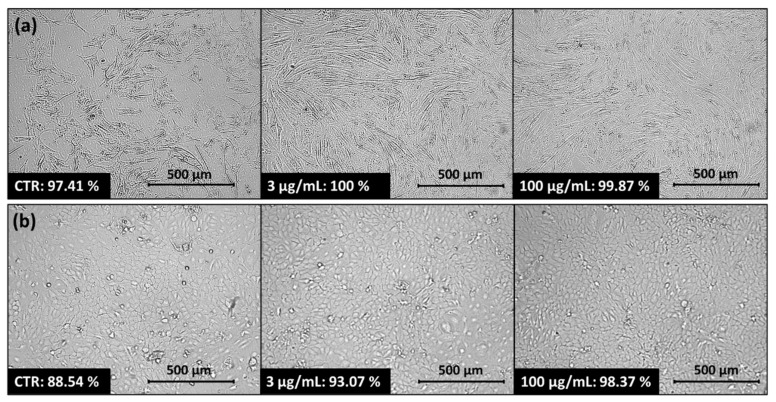
Confluency of (**a**) NHDF, (**b**) HaCaT cells without carrier (CTR) and after treatment with 3 µg/mL or 100 µg/mL solution of IIIc_FA.

A micellar carrier based on copolymer IIIc with encapsulated FA (IIIc_FA) was selected for further biological research. After applying IIIc_FA solution onto the normal human dermal fibroblasts (NHDF) and human epidermal keratinocyte (HaCaT) cell lines, an increase in cell confluence was observed after 72 h. This increase occurred with higher and lower concentrations, compared to the control cells (CTR) that were not treated with the carrier solution (Figure 7). The increase in confluence indicated that after applying the carrier, the cells retained their ability to proliferate, and even this process was enhanced by the delivery of the released active substance to the cells. A similar relationship was observed for two skin melanoma cell lines: malignant melanoma cells (Me45) and human metastatic melanoma variant of WM164 cell line (451-Lu). The application of IIIc_FA solution did not inhibit cell proliferation and cells multiplied to the level reached by the control cells or to an even higher level (Figure S9). In this case, although expected, it was not the desired effect because the obtained carriers should be inert to human cells and even improve their condition. Melanoma cell lines were studied comparatively.

**Figure 8:**
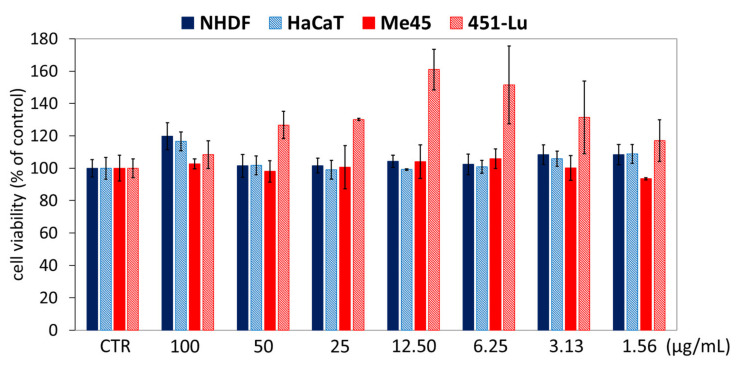
Cell viability after solution of IIIc_FA was added in different concentrations, followed by 72 h of incubation. Means ± S.D. from three independent experiments.

Moreover, cytotoxicity tests (MTT test) confirmed that the application of IIIc_FA solution did not decrease the cells’ viability compared to untreated controls, in which the viability was determined as 100% (Figure 8). Regardless of the solution concentration, the cell viability of NHDF, HaCaT and Me45 was higher than that of CTR by about 1–10%, while the highest increase in viability (10–60%) was observed for the 451-Lu cell line.

**Figure 9:**
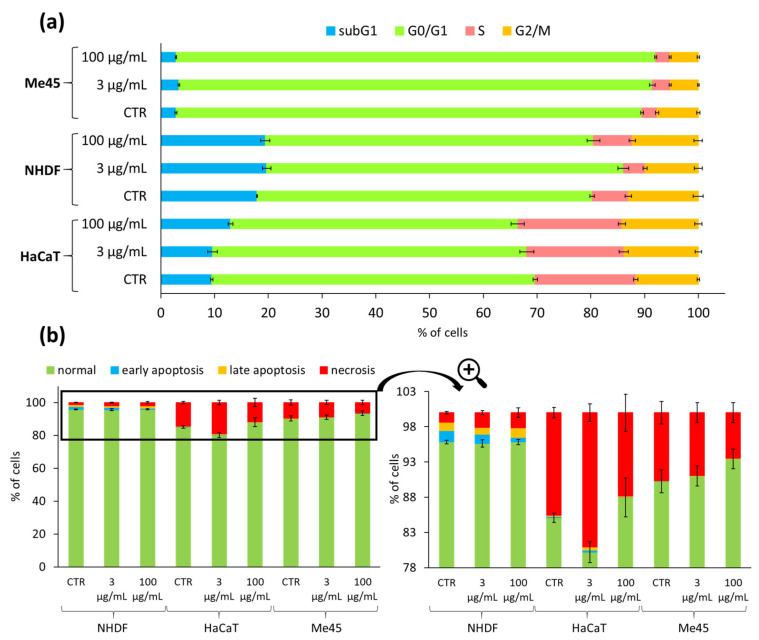
NHDF, HaCaT, and Me45 cell cycles, where subG1—dead cells, G0/G1—mononuclear cells, S—DNA replication, G2/M—mitosis (**a**), and results of Annexin V/propidium iodide (PI) double staining apoptosis assay in NHDF, HaCaT, and Me45 cells (**b**) after application of IIIc_FA solution (3 and 100 μg/mL), 72 h of incubation. Means ± S.D. from three independent experiments.

Additionally, the influence of IIIc_FA solution on the cell cycle of NHDF, HaCaT and tumor Me45 lines was examined. Cytometric evaluation indicated that IIIc_FA solution did not significantly alter the cell cycle phase distribution in comparison to the untreated control populations (CTR) after 72 h treatment, regardless of applied concentration (3 vs. 100 µg/mL) (Figure 9a). A slightly elevated subG1 cellular fraction (dead cells) in HaCaT was not considered to be significant because the difference was < 10% when compared to CTR. Cell cycle studies were also performed comparatively on one cancer cell line (Me45). Melanoma Me45 cells were arrested in G0/G1 phase, which may suggest a cytostatic potential of the active substances released from the carriers (Figure 9a). Although MTT tests did not show a decrease in Me45 cell viability, the released active substance may cause cell cycle arrest and a reduction in cell proliferation.

Analysis of the subG1 fraction of cells indicated the presence of a small number of damaged cells. Therefore, the Annexin-V test in combination with propidium iodide (PI) was performed for the characterization of cell death type induced by IIIc_FA solution (3 and 100 μg/mL). The results for NHDF cells do not indicate a high increase in necrotic or apoptotic states in comparison with CTR, regardless of the applied concentration (Figure 9b, Tables S3 and S4). Moreover, NHDF samples had a comparable number of necrotic cells and cells in the stage of early and late apoptosis, which meant the possibility of uncontrolled cell death was lower, and instead, these cells entered the apoptosis pathway. The highest share of dead cells was observed for HaCaT, which was also reflected in cell cycle tests, where HaCaT cells were characterized by the highest proportion of subG1 fraction (Figure 9b). However, when the obtained results were compared with CTR, the effect of IIIc_FA solution on the significant increase or decrease in the number of apoptotic or necrotic cells was not observed (15 vs. 12–19%). Moreover, after using a highly concentrated carrier solution, the number of dead cells slightly decreased compared to the control (15 vs. 12%), which may suggest a positive effect of released FA toward cell viability. A similar relationship as for HaCaT cells was observed for the Me45 tumor line. Moreover, for HaCat and Me45 cells, cell death occurred mainly through necrosis (total number of apoptotic cells in these cases was below 0.5%). In general, apoptosis is physiological cell death aimed at eliminating redundant, damaged or infected cells and does not cause inflammation, in contrast to necrosis, which is a passive, pathological process that affects whole groups of cells and causes inflammation. Despite the very high share of necrosis in HaCaT and Me45 cells, it also occurs in the corresponding control cells that were untreated with IIIc_FA; therefore, it was considered as a characteristic feature of a given cell line.

**Figure 10:**
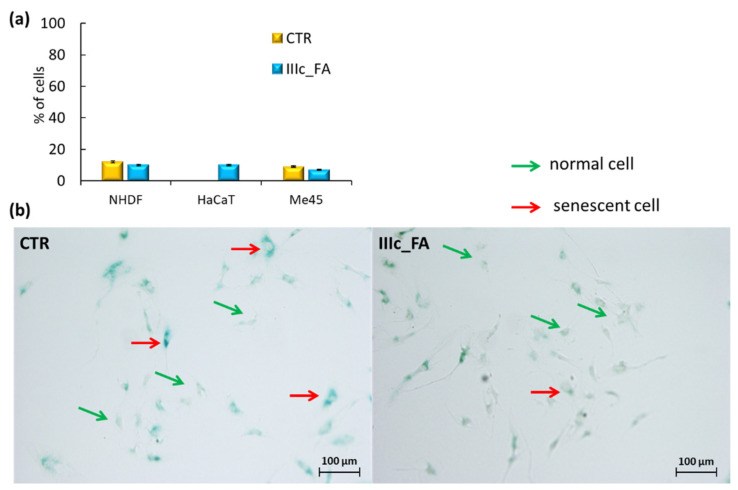
Senescence in cells after 72 h of incubation with IIIc_FA solution (100 µg/mL) (**a**) and microscopic images of NHDF normal and senescent cells in comparison to CTR cells (magnification 100×, transit channel, scale bars 100 µm) (**b**).

The last stage of determining the safe use of IIIc_FA in cosmetic products was achieved by the cell senescence test. The senescence effect induced by substances used in cosmetics is not desirable due to its association with the loss of cell dividing ability, changes in cell morphology, shape, physical appearance, and gene expression patterns. The performed assay was based on a histochemical stain for β-galactosidase activity at pH = 6, which was easily detectable in senescent cells (as blue-stained cells), but undetectable in quiescent or immortal cells (Figure 10, Figure S10). The obtained results did not display a significant increase in the share of senescent cells after treatment compared to control NHDF and Me45 cells (Figure 10). In the case of the HaCaT cell line, incubation of cells with IIIc_FA increased the number of senescent cells, but this effect was considered insignificant and safe.

## 3. Materials and Methods

### 3.1. Materials

Poly(ethylene glycol) methyl ether methacrylate (MPEGMA, M_n_ = 500 g/mol, 97%) was purchased from Aldrich (Poznań, Poland), and methanol (MeOH, 99%) and anisole (99%) were purchased from Alfa Aesar (Warsaw, Poland) and then were prepared as previously reported [41,42,43]. Copper (I) bromide (CuBr, Fluka, 98%, Steinheim, Germany) was purified according to reference [44]. Triethylamine (TEA, 99%), 4,4-dinonyl-2,2-dipyridyl (dNdpy, 97%), *N,N,N′,N”,N”-*pentamethyldiethylenetriamine (PMDETA, 98%), pyridine (99%), ethyl α-bromoisobutyrate (EiBBr, 98%), 2-bromoisobutyryl bromide (BriBuBr, 98%), poly(dimethylsiloxane) monohydroxy terminated (PDMS, M_n_ = 4670 g/mol), *L*-arginine (ARG, 98%), 0.1 M sodium phosphate buffer saline (PBS; pH = 7.4), 3-(4,5-dimethylthiazol-2-yl)-2,5-diphenyltetrazolium bromide (MTT), DMEM-F12 medium, trypsin, senescence cells histochemical staining kit, and Strat-M Membrane (Transdermal Diffusion Test Model, 25 mm) were purchased from Aldrich (Poznań, Poland). Sodium azide (NaN_3_, 99%) and ferulic acid (FA, 99%) were received from Acros (Geel, Belgium). *N,N*-Dimethylformamide (DMF, 99%), tetrahydrofuran (THF), anhydrous toluene (99%), L(+)-ascorbic acid (VitC, 99%), 2-propanol (98%), hydrochloric acid (HCl, 35–38%) from Chempur (Piekary Śląskie, Poland) were used as received. Physiological saline without Ca and Mg (PBS, PAA, Poland), Annexin-V apoptosis assay (BioLegend, San Diego, CA, USA), Annexin V Binding Buffer (BD Biosciences, San Jose, CA, USA), and propidium iodide solution (BD Biosciences, San Jose, CA, USA) were used as received. Normal Human Dermal Fibroblasts (NHDF) were purchased from Lonza (Lonza; Celllab, Warsaw, Poland). Malignant melanoma cells (Me45) and human metastatic melanoma variant of WM164 cell line (451-Lu) were obtained from the Maria Sklodowska-Curie Memorial Cancer Center and Institute of Oncology’s collection (Gliwice, Poland). Spontaneously immortalized human epidermal keratinocytes (HaCaT) were purchased from CSL Cell Line Service GmbH (Eppelheim, Germany). Additionally, 2-(Prop-1-en-2-carbonyloxy)ethyl hex-5-ynate (AlHEMA), 3,7-dimethyl-9-(2,6,6-trimethylcyclohex-1-en-1-yl)nona-2,4,6,8-tetraen-1-yl 2-bromo-2-methylpropanoate (RETBr) and 4-butyl-1,3-phenylene bis(2-bromo-2-methylpropanoate) (4nBREBr_2_) were prepared according to previous reports [41,42,44].

### 3.2. Characterization

^1^H- and ^13^C-NMR spectra were recorded using a UNITY/INOVA (Varian, Mulgrave, Victoria, Australia) spectrometer operating at 600 MHz or 300 MHz and 75 MHz, respectively, using dimethyl sulfoxide (DMSO-d_6_) or chloroform (CDCl_3_-d_1_) as solvent and tetramethylsilane (TMS) as an internal standard (*δ*_iso_(^1^H,^13^C) = 0 ppm). The monomer conversion was calculated based on gas chromatography (GC, Agilent Technologies 6850 Network GC System, Santa Clara, CA, USA) analysis, which was carried out in acetone. GC was equipped with a flame ionization detector. The temperature of the injector and detector was 250 °C, the initial and final temperature of the column was 40 °C and 200 °C, respectively. Monomer conversion was calculated via integration of signals at defined retention times for MPEGMA (1.8 min) and AlHEMA (10.0 min) that were compared to the retention time of anisole (4.9 min). Molecular weight (M_n_) and dispersity index (Đ) were determined by gel permeation chromatography (GPC, 1100 Agilent, Santa Clara, CA, USA) equipped with an isocratic pump, autosampler, degasser, thermostatic box for columns, and differential refractometer. The measurements were carried out in THF at 30 °C with a flow rate of 0.8 mL/min. GPC calculations were calibrated using linear polystyrene standards (580–300,000 g/mol). The critical micelle concentration (CMC) was measured by fluorescence spectrophotometry (FL, fluoroSENS Pro-11 spectrofluorimeter, CAMLIN, Lisburn, Ireland), using pyrene as a fluorescence probe. Excitation spectra of pyrene (λ = 390 nm) were recorded at a constant concentration (3.0 × 10^−4^ mol/L) and polymer concentrations in the range of 5 × 10^−4^ to 1.0 mg/mL. Particle sizes and distributions, i.e., apparent hydrodynamic diameter (D_h_) and polydispersity index (PDI), were measured in PMMA cells at 25 °C using dynamic light scattering (DLS, Zetasizer Nano-S90, Zetasizer Software, Malvern Technologies, Malvern, PA, USA) equipped with a He-Ne laser at a fixed scattering angle (173°). Because of the signal changes on the detector caused by constantly vibrating particles (the Brownian motions), the speed of particle movement is measured and then converted to particle size distribution using the Stokes–Einstein equation. Molecular or translational diffusion of micelles is related to diffusion coefficients. The measurements were carried out on two samples from three independent runs to obtain an average value. Ultraviolet-visible light spectroscopy (UV-Vis, Thermo Fisher Scientific Evolution 300, Waltham, MA, USA) was used to determine the amount of entrapped and released active substance over time. The measurements were carried out in PMMA cells. In vitro, active substance permeation was evaluated using Franz diffusion cells (Teledyne Hanson Research, Phoenix DB-6, Variel Ave, Chatsworth, CA, USA) and transdermal diffusion test model membranes mimicking artificial skin. The absorbance of the formazan product created during the MTT test was determined at 570 nm using a microplate reader (Epoch; BioTek, Winooski, VT, USA). Cytometric analyses were performed using an Aria III flow cytometer (Becton Dickinson; Franklin Lakes, NJ, USA) with the fluorescein isothiocyanate (FITC) configuration (488 nm excitation; emission: longpass filter (LP) mirror 503, bandpass filter (BP) 530/30) or phycoerythrin (PE) configuration (547 nm excitation; emission: 585 nm) and at least 10,000 cells were counted. Automated cell confluence analysis and the monitoring of cell density and viability were carried out using Live Cell Analyzer, JuLI™ Br (NanoEnTek Inc., Seoul, Korea).

### 3.3. Synthesis of P(AlHEMA-co-MPEGMA) with RETBr as Initiator (Example for II)

In a Schlenk flask, the following substances were added under nitrogen atmosphere: RETBr (19.42 mg, 0.045 mmol), dNdpy (41.00 mg, 0.100 mmol), monomers: MPEGMA (6.20 mL, 13.39 mmol), AlHEMA (1.0 mL, 4.47 mmol), and MeOH (0.180 mL) and anisole (0.540 mL) as solvents. The total volume of solvents accounted for 10% of the volume of monomers. The mixture was degassed by three freeze–pump–thaw cycles and CuBr (6.40 mg, 0.045 mmol) was added. The reaction was carried out at 60 °C under a constant flow of inert gas. In order to quench the polymerization reaction, the Schlenk flask was opened and exposed to air. Subsequently, the mixture was dissolved in chloroform, passed through a neutral alumina column to remove CuBr, and the obtained solution was concentrated. The product was precipitated in diethyl ether and dried under vacuum. The synthesis procedures of P(AlHEMA-*co*-MPEGMA) copolymers using EiBBr or 4nBREBr_2_ as an initiator are described in Supplementary Materials (Synthesis procedure S1,2).

### 3.4. Preparation of Azide Derivative PDMS-N_3_ via PDMS-OH Modification

PDMS-OH (9.70 g, 2.08 mmol) was dissolved in chloroform (80 mL) to yield a colorless solution.

Then, TEA (0.32 mL, 2.30 mmol) was added dropwise and the reactor was cooled to 0 °C in an ice/water bath, followed by the addition of BriBuBr (0.28 mL, 2.30 mmol). The reaction mixture was stirred for 24 h at room temperature and without light. The mixture was transferred into a separator with chloroform and extracted consecutively with H_2_O (2 × 100 mL). The organic phase was concentrated. The product (PDMS-Br) was precipitated in MeOH and dried under vacuum to constant mass. Yield: 94%. ^1^H NMR (600 MHz, CDCl_3_, ppm): 3.80 (2H, -CH_2_O(O=)C-), 3.61 (2H, -OCH_2_-), 3.52 (2H, -OCH_2_-), 2.10 (6H, -C(CH_3_)_2_Br), 1.69 (2H, -CH_2_CH_2_CH_2_-), 1.38 (9H, -C(CH_3_)_3_), 0.95 (2H, -Si(CH_3_)_2_-CH_2_-), 0.60 (6H, -Si(CH_3_)_2_-CH_2_-), 0.12 (6H, -Si(CH_3_)_2_-C(CH_3_)_3_ and n * 6H, -[Si(CH_3_)_2_]_n_-).

PDMS-Br (8.00 g, 1.70 mmol) and NaN_3_ (110.70 mg, 1.70 mmol) were dissolved in anhydrous THF (80 mL). The reaction was carried out for 24 h at room temperature and without light. The reaction work-up and purification were performed according to the above-described procedure. The product (PDMS-N_3_) was dried under vacuum to constant mass. Yield: 87%. ^1^H NMR (600 MHz, CDCl_3_, ppm): 3.80 (2H, -CH_2_O(O=)C-), 3.61 (2H, -OCH_2_-), 3.52 (2H, -OCH_2_-), 2.02 (6H, -C(CH_3_)_2_N_3_), 1.69 (2H, -CH_2_CH_2_CH_2_-), 1.38 (9H, -C(CH_3_)_3_), 0.95 (2H, -Si(CH_3_)_2_-CH_2_-), 0.60 (6H, -Si(CH_3_)_2_-CH_2_-), 0.14 (6H, -Si(CH_3_)_2_-C(CH_3_)_3_ and n * 6H, -[Si(CH_3_)_2_]_n_-). ^13^C NMR (75 MHz, CDCl_3_, ppm): 202 (C11, -COO-), 74 (C8, C9, -CH_2_-O-CH_2_-), 67 (C12, C(CH_3_)_2_N_3_), 66 (C10, -CH_2_-O(O=)C-), 32 (C7, Si(CH_3_)_2_CH_2_CH_2_-), 31 (C6, -Si(CH_3_)_2_CH_2_CH_2_-), 23 (C1, -C(CH_3_)_3_), 22 (C13, -C(CH_3_)_2_N_3_), 18 (C2, -C(CH_3_)_3_), 1 (C3, C4, -[Si(CH_3_)_2_]_n_-).

### 3.5. “Click” Reaction (CuAAC, Example for Ic)

To a solution of polymer I (1.00 g, 9.73 × 10^−3^ mmol containing 0.566 mmol of AlHEMA units) in THF (20 mL), PDMS-N_3_ (2.66 g, 0.566 mmol) and 2.5-fold molar excess of PMDETA (0.30 mL, 1.415 mmol) were added. The reaction mixture was degassed under a constant flow of inert gas for 30 min. Subsequently, CuBr (202.80 mg, 1.415 mmol) was added and the reaction was completed after 48 h (constant mixing, room temperature, darkness). CuBr was removed by cationite (Dowex) and the obtained solution was concentrated. The product was precipitated in MeOH and dried to constant mass.

### 3.6. Encapsulation and Release Studies

The amphiphilic heterograft copolymer (100 mg) and active substance (the weight ratio of copolymer: active substance = 1:1) was dissolved in MeOH (15 mL), and H_2_O was added dropwise (200 vol% of MeOH) ensuring constant mixing. The encapsulation process took place over a 24 h period. Subsequently, the organic solvent was evaporated and the unloaded active substance was separated by centrifugation (6000 rpm/5 min, 24 °C). The aqueous fraction was lyophilized by freezing to obtain a solid product. To determine the amount of encapsulated substance, a solution of loaded micelles in PBS was prepared (at a given concentration) and calculated using UV-Vis (resolution > 2.0 at 0.5 nm spectral bandwidth, SBW) analysis. The absorbance of active substances was measured at λ = 310 nm for FA, λ = 306 nm for ARG, and λ = 267 nm for VitC. Drug loading content (DLC) was calculated as the percentage ratio of weight of drug loaded into the micelle to weight of total polymer and loaded drug.

The solution of loaded micelles in PBS (pH = 7.4 or 5.5, 1.0 mg/mL) was placed into a dialysis membrane bag (cellulose, MWCO = 3.5 kDa) and incorporated into a vial with PBS (50 mL, pH = 7.4 or 5.5). The release process was performed in a water bath (37 °C), ensuring constant mixing. The concentration of the released drug was calculated by the absorbance of the released medium samples and was measured by UV-Vis spectroscopy at the wavelength characteristic for each active substance.

### 3.7. Permeation Tests in Franz Diffusion Cells

PBS solution (15 mL) was introduced into a diffusion cell (acceptor chamber) equipped with a magnetic stirrer. The membrane and donor chamber were placed and the test carrier solution (1.8 mL, 1.0 mg/mL) was introduced into the donor chamber. The experiment was carried out at the temperature of 37 °C, maintaining continuous stirring (V = 400 rpm) for 24 h. During analysis, 200 µL of the solution was extracted from the acceptor chamber at specified intervals, and then the diffusion cell was supplemented with the same amount of PBS. The collected samples were subjected to UV-Vis analysis. Flow through the membrane (*J*) was calculated using the following equations:

(1) $$HLB=20*\;\frac{M_{hphil.}}{M_{n}}$$

(2) $$D=\frac{e^{2}}{6*t}$$

(3) $$J=\frac{D*HLB*\Delta c}{e}$$

where *HLB*—hydrophilic/lipophilic balance, *M_hphil._—*molecular weight of the hydrophilic fraction, *M_n_*—molecular weight of copolymer, *D*—diffusion coefficient, *e*—membrane thickness, *t*—lag time, *J*—flow through the membrane, Δc—concentration difference on both sides of the membrane.

### 3.8. MTT Cytotoxicity Assay

For MTT assays, cells (Procedure S3) were plated 24 h before drug treatment onto 96-well plates at 10,000 cells/well in 0.2 mL medium. Appropriate controls, DMSO in the fresh medium, were prepared. A series of suspension dilutions (1.563–100 µg/mL) were added into wells. Cells were incubated with compounds for 72 h, and then the solutions were removed and incubated with MTT solution (50 μL of 0.5 mg/mL in Roswell Park Memorial Institute medium (RPMI) 1640 without phenol red) for 2–3 h. After removing MTT solution, the formazan crystals were dissolved in 75 μL of isopropanol/HCl (*v*/*v* = 1/0.04) mixture. The absorbance of the formazan product was measured at 570 nm using a microplate reader. The experiment was conducted in three independent repetitions, with six technical repeats for each tested concentration, and results were expressed as a survival fraction [%] of the control.

### 3.9. Apoptosis and Cell Cycle Analyses by Flow Cytometry

The fraction of dead cells was detected 72 h after treatment with the studied compound using an Annexin-V apoptosis assay and propidium iodide (PI) solution (100 μg/mL) uptake test. Cells collected from the plates after centrifugation (3 min, 0.6 g, room temperature) and supernatant removal were suspended in 50 μL of cold Annexin-V labeling buffer and stained with FITF-labeled Annexin-V antibody for 20 min (2.5 μL). Next, cells were incubated in the dark with 10 μL of PI for 20 min. A total of 250 μL of Annexin-V labeling buffer was then added and samples were incubated in the dark on ice for 15 min. Cytometric analyses were performed immediately using an Aria III flow cytometer and at least 10,000 cells were counted. For cell cycle analysis, the cells were stained with 250 μL of hypotonic buffer (comprised from PI 100 μg/mL in PBS; 5 mg/L of citric acid; 1:9 Triton-X solution; RNase 100 μg/mL in PBS from Sigma) and DNA content was assessed by fluorescence measurements.

### 3.10. Cell Senescence Tests

For the cell senescence test, cells were plated 24 h before drug treatment at 10,000 cells/well in 2 mL medium. Then, the growth medium was aspirated from the cells, and for the next 72 h replaced with medium containing tested compounds at a dose of 100 μg/mL. After standard incubation, the cells were washed twice with 1 mL of PBS per well. Carefully, the entire wash solution was removed by aspiration, so the cells did not detach. A total of 1.5 mL per well of Fixation Buffer (prepared according to the producer protocol, Sigma, Poznan, Poland) was added and the plate was incubated for 6–7 min at room temperature. Then, the cells were rinsed 3 times with PBS (1 mL) and the Staining Mixture (1 mL) was added. The cells were incubated at 37 °C without CO_2_ until they were stained blue (24 h). The cells were observed under a microscope and blue-stained cells and total number of cells were counted. The percentage of cells expressing β-galactosidase (senescent cells) was calculated.

## 4. Conclusions

The graft copolymers P(AlHEMA-*co*-MPEGMA) were obtained for a further “click” reaction with an azide derivative of PDMS. The PDMS grafting was completed with an efficiency of 33–47%. The self-assembling behavior of the amphiphilic PEG/PDMS hete-rografted copolymers in aqueous solution was employed to encapsulate ARG, FA or VitC with a relatively high efficiency (DLC > 50%), except for micelles with VitC (DLC = 2–62%). In vitro studies carried out in PBS solution (pH = 7.4 or 5.5) demonstrated that the maximum release of active substances was reached after 60–180 min depending on the system. The effectiveness of the release process of encapsulated substances at neutral or slightly acidic pH was dependent on their hydrophilicity and acid-base properties. The diffusion tests carried out in Franz cells showed that the release of active substances was mainly retained in the membrane acting as artificial skin and diffused slightly into the solution (2–16%). The larger particles (>300 nm) did not exclude these systems from potential application in cosmetology as they could be used in the form of masks, where a carrier does not diffuse through the skin. Additionally, the released substance could penetrate into the skin or act on its surface. A beneficial property was no negative effects of the selected carrier on cell viability (>100%) and proliferation capacity, as well as no impact on cell apoptosis/necrosis or cell cycle interruption. The performed senescence test did not show induction of this pathway, which additionally confirmed that the use of the tested systems on human skin is potentially safe. Thus, these physicochemical and biological studies evaluated the possibility of safe use of the test carrier with FA, due to the lack of toxic effect on the selected cell lines compared to the control cells. The presented micellar systems are good candidates for the purpose of carrying cosmetic substances in products, such as masks and eye pads. Despite the satisfactory results of the conducted research, further specific biological assessments are required for the final verification of the applicability of the designed heterograft copolymers in cosmetology.

## Figures and Tables

**Table 1:** Data of AlHEMA/MPEGMA copolymers synthesized by atom transfer radical polymerization (ATRP).

No.	M1/M2	Time (h)	DP_n_^a^	M_n_^a.^ (g/mol)	M_n_^b.^ (g/mol)	D ^b^
I	25/75	2.5	238	102,500	54,400	1.29
II	25/75	4.0	173	76,600	61,700	1.44
III	50/50	3.0	158	59,600	57,000	1.76
IV	25/75	24.0	183	71,800	26,200	1.28
V	50/50	4.0	182	58,200	47,600	1.88

Conditions: [M1+M2]_0_/[In]_0_/[CuBr]_0_/[dNdpy]_0_ = 400/1/1/2.25, anisole/methanol = 9:1 10 vol% mon; 60 °C, where: M1 is AlHEMA and M2 is MPEGMA, In is EiBBr (I), RETBr (II–III), 4nBREBr_2_ (IV–V), DP_n_—polymerization degree, M_n_—molecular weight, D—dispersity index, ^a^ calculated using conversion determined by gas chromatography (GC) analysis; ^b^ determined by gel permeation chromatography (GPC) in tetrahydrofuran (THF) with polystyrene standards.

**Table 2:** Data for modifications of PDMS.

Polymer	DP_n_^a^	M_n,NMR_^a^ (g/mol)	M_n,GPC_^b^ (g/mol)	D ^b^
**PDMS-OH**	65	5100	5550	1.11
**PDMS-Br**	65	5200	5800	1.11
**PDMS-N_3_**	65	5170	5400	1.14

^a^ calculated by NMR analysis, ^b^ determined by GPC in THF with polystyrene standards.

**Table 3:** Characteristics of “click” graft copolymers P((HEMA-*graft*-PDMS)-*co*-MPEGMA).

No.	F_AlHEMA_ (%)	E_click_^a^ (%)	n_tria_^a^	DG_PDMS_ (%)	DG (%)	F_hphil._ (wt.%)	M_n_^a^ (g/mol)	D ^b^	CMC ^c^ (mg/mL)
Ic	24	33	19	8	84	44	201,500	1.21	0.2044
IIc	20	40	14	8	88	46	149,700	1.13	0.2055
IIIc	44	37	26	17	72	22	195,300	1.44	0.0359
IVc	39	47	33	18	79	23	244,000	1.16	0.0348
Vc	66	43	52	28	63	9	329,200	1.22	0.0063

F_AlHEMA_—content of AlHEMA in the copolymer; E_click_—efficiency of “click” reaction calculated by the following equation E_click_ = (H_f_/(H_f_ + H_≡__CH_)) * 100%, where H_f_—integral area of signals from CH proton of triazole ring and H_≡__CH_—alkyne proton from unreacted AlHEMA units; n_tria._—number of triazole rings in the copolymer related to number of PDMS side chains; DG_PDMS_—degree of PDMS grafting (n_tria_/DP_n_); DG—degree of grafting, including both types of side chains ((DP_MPEGMA_ + n_tria._)/DP_n_); F_hphil._—content of polyethylene glycol methyl ether (MPEG) in copolymer; ^a^ calculated by NMR analysis, ^b^ determined by GPC, ^c^ determined by fluorescence spectrophotometry.

**Table 4:** D_h_ of the polymeric micelles.

No.	D_h_^a^ ± SD (nm)/PDI			
	Empty	VitC	ARG	FA
Ic	154 ± 25/0.232	460 ± 72/0.198	254 ± 9/0.616	661 ± 31/0.096
IIc	229 ± 41/0.579	^b^ 604 ± 30/0.629	345 ± 66/0.460	167 ± 25/0.0283
IIIc	397 ± 23/0.012	^b^ 238 ± 40/0.377	248 ± 26/0.868	^b^ 258 ± 24/0.424
IVc	361 ± 15/0.350	152 ± 11/1.000	305 ± 48/0.352	145 ± 6/0.226
Vc	185 ± 4/0.354	^b^ 216 ± 46/0.361	321 ± 21/0.412	^b^ 9 ± 1/1.000

^a^ D_h_ by intensity, ^b^ value of particle size for dominated fraction.

**Table 5:** Data for permeation tests in Franz chamber cells.

No.	DLC (%)	DLC_apr_ (%)	^FC^R_max_ (%)	T_max_ (min)	$D\;(\frac{cm^{2}}{h})\;*\;10^{-4}$	HLB	$J\;\left(\frac{\frac{µg}{cm^{2}}}{h}\right)$
IIIc_VitC	19	1	95	30	9.0	2.91	0.76
IIIc_FA	83	26	69	240	9.0	2.91	22.81
IVc_VitC	44	1	98	60	9.0	4.55	1.04
IVc_FA	77	25	68	10	9.0	4.55	33.81

DLC—drug loading content in carrier before permeation test, DLC_apr_—drug loading content in carrier after permeation test, ^FC^R_max_—amount of released substance using Franz cells, T_max_—time of permeation test after which the concentration of the substance in the solution did not increase, D—diffusion coefficient, HLB—hydrophilic/lipophilic balance, J—flow through the membrane.

## Data Availability

Not applicable.

## Supplementary Materials

The following are available online at https://www.mdpi.com/1422-0067/22/3/1202/s1. Synthesis procedures: P(AlHEMA-*co*-MPEGMA) with EiBBr or 4nBREBr_2_ as Initiator. Cell culture. Tables: D_h_ by volume for obtained micelles. Maximum amount of released drug for pH = 7.4 ^a^ and pH = 5.5 ^b^ in time. Results and typical graphs of Annexin V/PI double staining apoptosis assay. Figures: ^1^H NMR spectrum of the reaction mixture for copolymerization I. GPC traces of representative AlHEMA/MPEGMA copolymers. ^1^H and ^13^C NMR spectra of PDMS-OH and its derivatives. GPC traces before and after modifications of PDMS. Plots of intensity I_336_/I_332_ ratio as a function of the logarithm of copolymer concentration in aqueous solution determined by spectrofluorometry. Size distribution intensity plots for micelles formed by heterografted copolymers Ic, IIIc, and Vc. Kinetic profiles for VitC and FA released from heterografted polymer micelles in PBS, pH = 5.5. Increase in confluency of Me45, 451-Lu cells in time treated with copolymer IIIc_FA (c = 100 µg/mL). Me45 normal and senescent cells observed under the microscope after senescence test.
