# Supplementary material for: Micellar Carriers of Active Substances Based on Amphiphilic PEG/PDMS Heterograft Copolymers: Synthesis and Biological Evaluation of Safe Use on Skin
Source: Int J Mol Sci. 2021 Jan 26;22(3):1202. doi: 10.3390/ijms22031202 (PMC7865956; doi:10.3390/ijms22031202)
Supplement: Supplementary file 1 [file ijms-22-01202-s001.pdf]

Supporting Information

Micellar carriers of active substances based on amphiphilic PEG/PDMS heterograft copolymers: synthesis and biological evaluation of safe use on skin

Justyna Odrobińska[1], Magdalena Skonieczna[2,3], and Dorota Neugebauer [1,*]

[1] Department of Physical Chemistry and Technology of Polymers, Faculty of Chemistry, Silesian University of Technology, 44-100 Gliwice, Poland
[2] Department of Systems Biology and Engineering, Silesian University of Technology, Akademicka 16, 44-100 Gliwice, Poland
[3] Biotechnology Centre, Silesian University of Technology, Krzywoustego 8, 44-100 Gliwice, Poland
* Correspondence: dorota.neugebauer@polsl.pl

Content:

Synthesis procedure S1. Synthesis of P(AlHEMA-*co*-MPEGMA) with EiBBr initiator (Example for I).
Synthesis procedure S2. Synthesis of P(AlHEMA-*co*-MPEGMA) with 4nBREBr$_2$ initiator (Example for IV).
Procedure S3. Cell culture.

Table S1. D_h by volume for obtained micelles.
Table S2. Maximum amount of released drug for pH=7.4[a] and pH=5.5[b] in time.
Table S3. Results of Annexin V/PI double staining apoptosis assay.
Table S4. Typical graphs of Annexin V/PI double staining apoptosis assay.

Figure S1. ^{1}H NMR spectrum of the reaction mixture for copolymerization I, where m, p - the resonances related to monomer and polymer, respectively.
Figure S2. GPC traces of representative AlHEMA/MPEGMA copolymers.
Figure S3. ^{1}H NMR spectra of (a) PDMS-OH, (b) PDMS-Br and (c) PDMS-N$_3$.
Figure S4. ^{13}C NMR spectra of (a) PDMS-OH, (b) PDMS-N$_3$.
Figure S5. GPC traces before and after modifications of PDMS.
Figure S6. Plots of intensity I_{336}/I_{332} ratio as a function of the logarithm of copolymer concentration in aqueous solution determined by spectrofluorometry.
Figure S7. Size distribution intensity plots for micelles formed by heterografted copolymers (a) Ic, (b) IIIc, and (c) Vc.
Figure S8. Kinetic profiles for (a) VitC, and (b) FA released from heterografted polymer micelles in PBS, pH=5.5.
Figure S9. Increase in confluency of (a) Me45, (b) 451-Lu cells in time treated with copolymer IIIc_FA (c = 100 μg/mL), CTR is control.
Figure S10. Me45 normal and senescent cells observed under the microscope after senescence test. Magnification 100 x, transit channel, scale bars 100 μm.

Synthesis procedure S1. Synthesis of P(AlHEMA-*co*-MPEGMA) with EiBBr Initiator (Example for I).

dNdpy (41.05 mg, 0.101 mmol), MPEGMA (6.20 mL, 13.39 mmol), AlHEMA (1.00 g, 4.46 mmol), and solvents (10 vol.% of monomers; MeOH : ANS = 1: 6): MeOH (0.103 mL), ANS (0.612 mL) were placed in a Schlenk flask and degassed by two freeze–pump–thaw cycles. Then, EiBBr (6.62 μL, 0.045 mmol) was added and degassed again. After that, CuBr (6.40 mg, 0.045 mmol) was added. The reaction flask was immersed in an oil bath at 60 °C. The polymerization was stopped by exposure to air. Then, the mixture was dissolved in chloroform and passed through a neutral alumina column to remove CuBr. The solution was concentrated and the polymer was precipitated by dropwise addition of a concentrated solution into diethyl ether. The product was isolated by decantation and dried under vacuum to constant mass.

Synthesis procedure S2. Synthesis of P(AlHEMA-*co*-MPEGMA) with 4nBREBr$_2$ Initiator (Example for IV).

4nBREBr$_2$ (22.10 mg, 0.051 mmol), dNdpy (41.05 mg, 0.101 mmol), MPEGMA (6.20 mL, 13.39 mmol), AlHEMA (1.00 g, 4.47 mmol), and solvents (10 vol.% of monomers; MeOH : ANS = 1: 3): MeOH (0.180 mL), ANS (0.540 mL) were placed in a Schlenk flask and then degassed by three freeze–pump–thaw cycles. After that, CuBr (6.40 mg, 0.045 mmol) was added. The reaction flask was immersed in an oil bath at 60 °C. The next steps were performed according to above-described procedure for the synthesis of P(AlHEMA-*co*-MMA) with EiBBr (Synthesis procedure S1).

Procedure S3. Cell culture.

All cells (Me45, 451-Lu, NHDF, HaCaT) were grown in sterile culture bottles with a culture area of 75 cm^2 in DMEM-F12 medium supplemented with 10% (v/v) inactivated fetal bovine serum (FBS) (EURx, Poland) and 1% antibiotics (10,000 μg/mL of streptomycin and 10,000 units/mL of penicillin) (Sigma-Aldrich, Germany) at 37 °C in a humidified atmosphere with 5% CO_2. Cell lines were seeded in a 96-well plate at a density of 10,000 cells per well in the case of MTT tests and a density of 100000 cells per well in the case of apoptosis and cell cycle analyses (6-well plate).

Table S1. D_h by volume for obtained micelles.

No.	D_h ± SD (nm)			
	**empty**	**VitC**	**ARG**	**FA**
Ic	154±21	543±70	260±4	690±31
IIc	[a]64±17	[a]92±8	[a]117±2	134±4
IIIc	431±98	267±55	231±27	[a]50±8
IVc	385±9	142±13	458±82	105±3
Vc	93±10	178±26	364±20	10±1

[a] value of particle size for dominated fraction

Table S2. Maximum amount of released drug for pH=7.4[a] and pH=5.5[b] in time.

No.	Maximum amount of released drug (%)/time (min)					
	VitC[a]	VitC[b]	ARG[a]	ARG[b]	FA[a]	FA[b]
Ic	43/60	77/75	23/10	n.o.	95/90	80/180
IIc	63/130	63/180	74/60	n.o.	84/240	69/180
IIIc	31/130	13/120	92/180	n.o.	99/120	76/180
IVc	24/50	99/75	96/180	n.o.	92/300	53/180
Vc	24/80	59/50	n.o.	n.o.	81/300	82/180

n.o.: no released substance was observed

Table S3. Results of Annexin V/PI double staining apoptosis assay.

IIIc_FA concentration [μg/mL]		% of cells ± S.D.			
		A-/PI-	A+/PI-	A+/PI+	A-/PI+
0 (CTR)	NHDF	95.82 ± 0.24	1.64 ± 0.15	1.17 ± 0.13	1.36 ± 0.18
3		95.61 ±0.51	1.34 ± 0.18	0.96 ± 0.30	2.09 ± 0.26
100		95.83 ± 0.38	0.64 ± 0.14	1.36 ± 0.29	2.17 ± 0.66
0 (CTR)	HaCaT	85.07 ± 0.67	0.19 ± 0.06	0.17 ± 0.04	14.58 ± 0.72
3		80.21 ± 1.48	0.24 ± 0.16	0.46 ± 0.11	19.09 ± 1.27
100		87.94 ± 2.76	0.05 ± 0.08	0.19 ± 0.13	11.81 ± 2.65
0 (CTR)	Me45	90.22 ± 1.63	0.03 ± 0.02	0.07 ± 0.03	9.68 ± 1.60
3		91.00 ± 1.44	0.01 ± 0.01	0.07 ± 0.03	8.91 ± 1.42
100		93.43 ± 1.42	0.02 ± 0.02	0.06 ± 0.03	6.49 ± 1.41

A−/PI−: live cells; A+/PI−: early apoptosis; A+/PI+: late apoptosis; A−/PI+: necrosis

Table S4. Typical graphs of Annexin V/PI double staining apoptosis assay.

IIIc_FA concentration [μg/mL]	typical apoptosis dot plots		
	0 (CTR)	3	100
NHDF	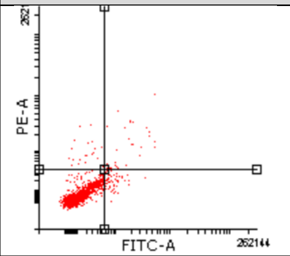	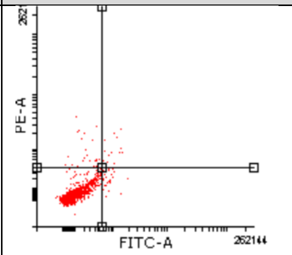	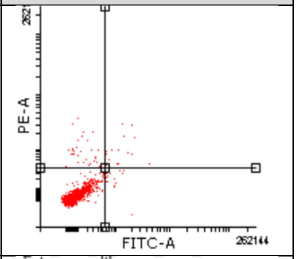
HaCaT	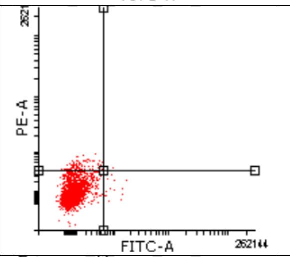	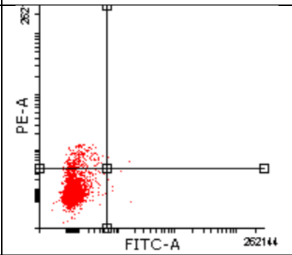	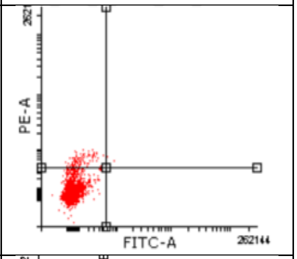
Me45	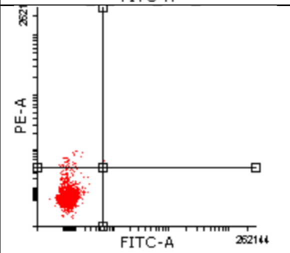	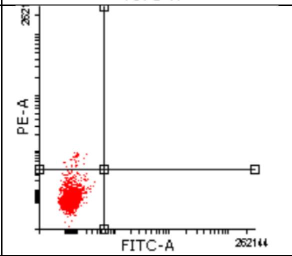	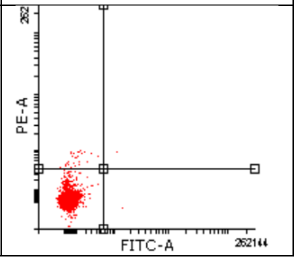

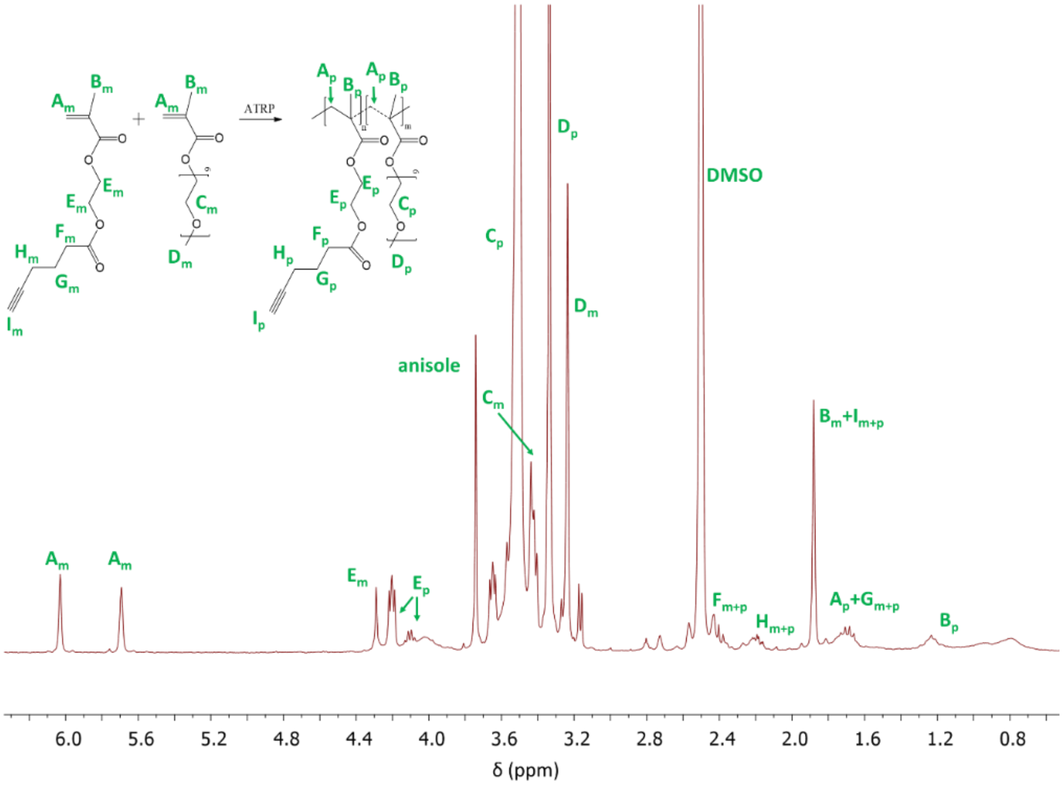

Figure S1. ^{1}H NMR spectrum of the reaction mixture for copolymerization I, where *m, p* - the resonances related to monomer and polymer, respectively.

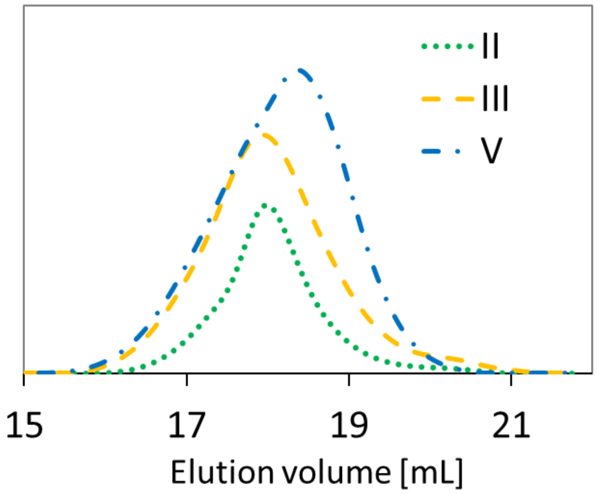

Figure S2. GPC traces of representative AlHEMA/MPEGMA copolymers.

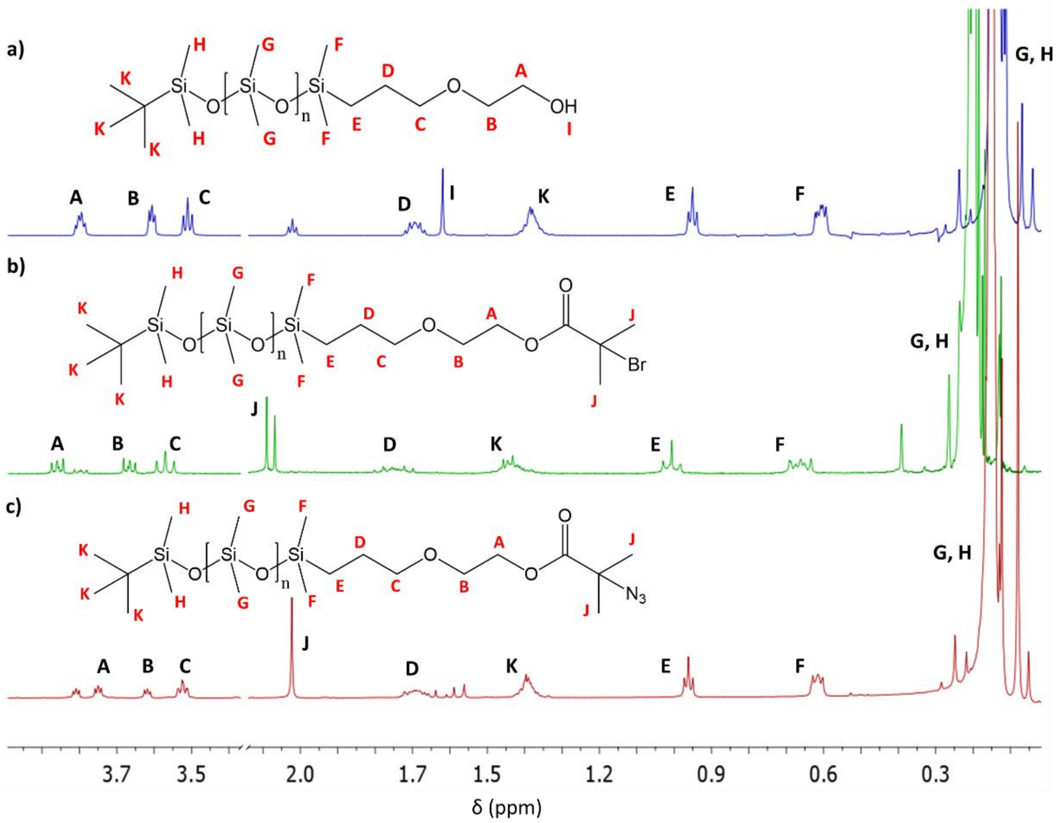

Figure S3. ^{1}H NMR spectra of (a) PDMS-OH, (b) PDMS-Br and (c) PDMS-N_3.

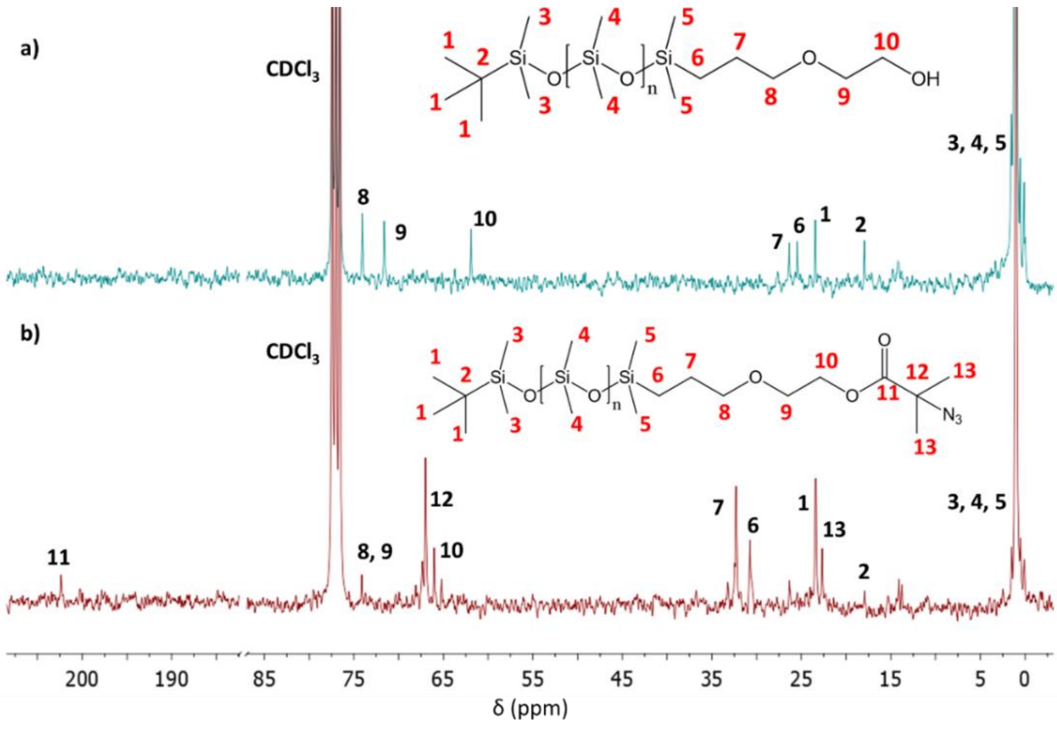

Figure S4. ^{13}C NMR spectra of (a) PDMS-OH, (b) PDMS-N_3.

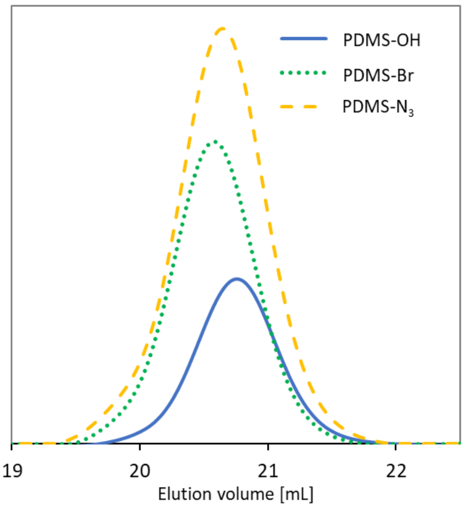

Figure S5. GPC traces before and after modifications of PDMS.

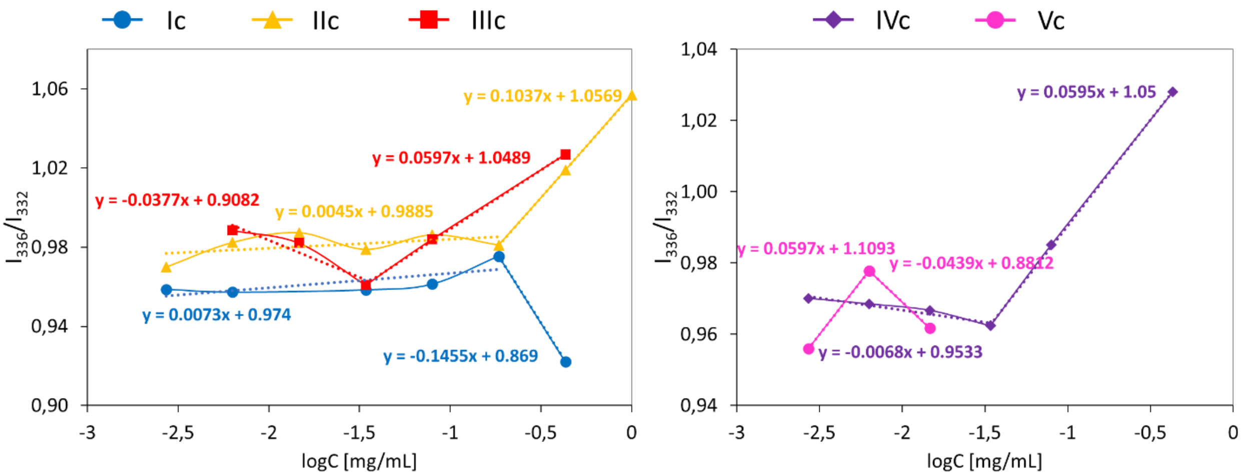

Figure S6. Plots of intensity I_{336}/I_{332} ratio as a function of the logarithm of copolymer concentration in aqueous solution determined by spectrofluorometry.

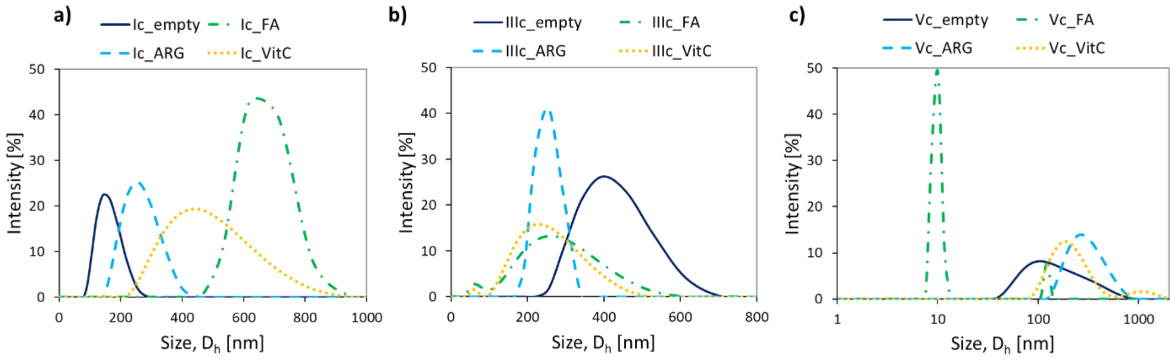

Figure S7. Size distribution intensity plots for micelles formed by heterografted copolymers (a) Ic, (b) IIIc, and (c) Vc.

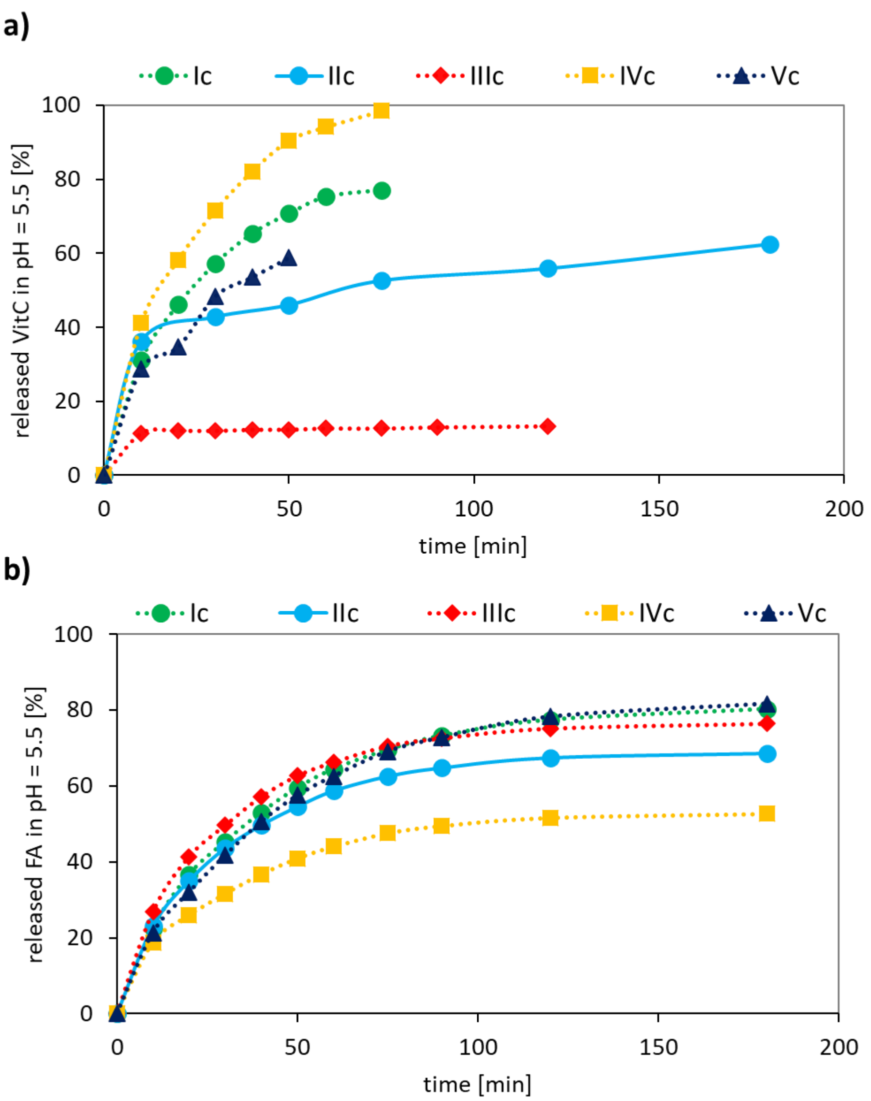

Figure S8. Kinetic profiles for (a) VitC, and (b) FA released from heterografted polymer micelles in PBS pH=5.5.

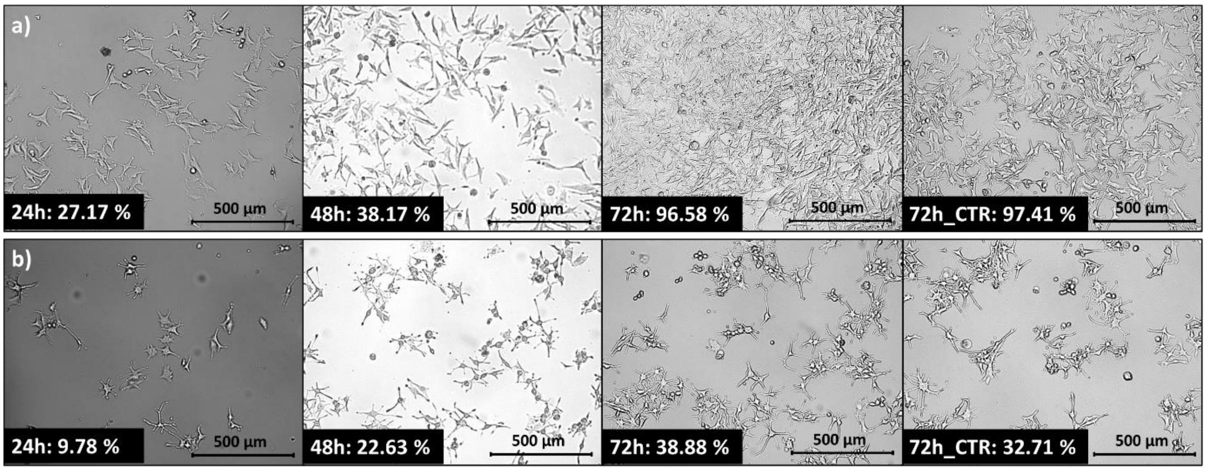

Figure S9. Increase in confluency of (a) Me45, (b) 451-Lu cells in time treated with copolymer IIIc_FA (c = 100 µg/mL), CTR is control.

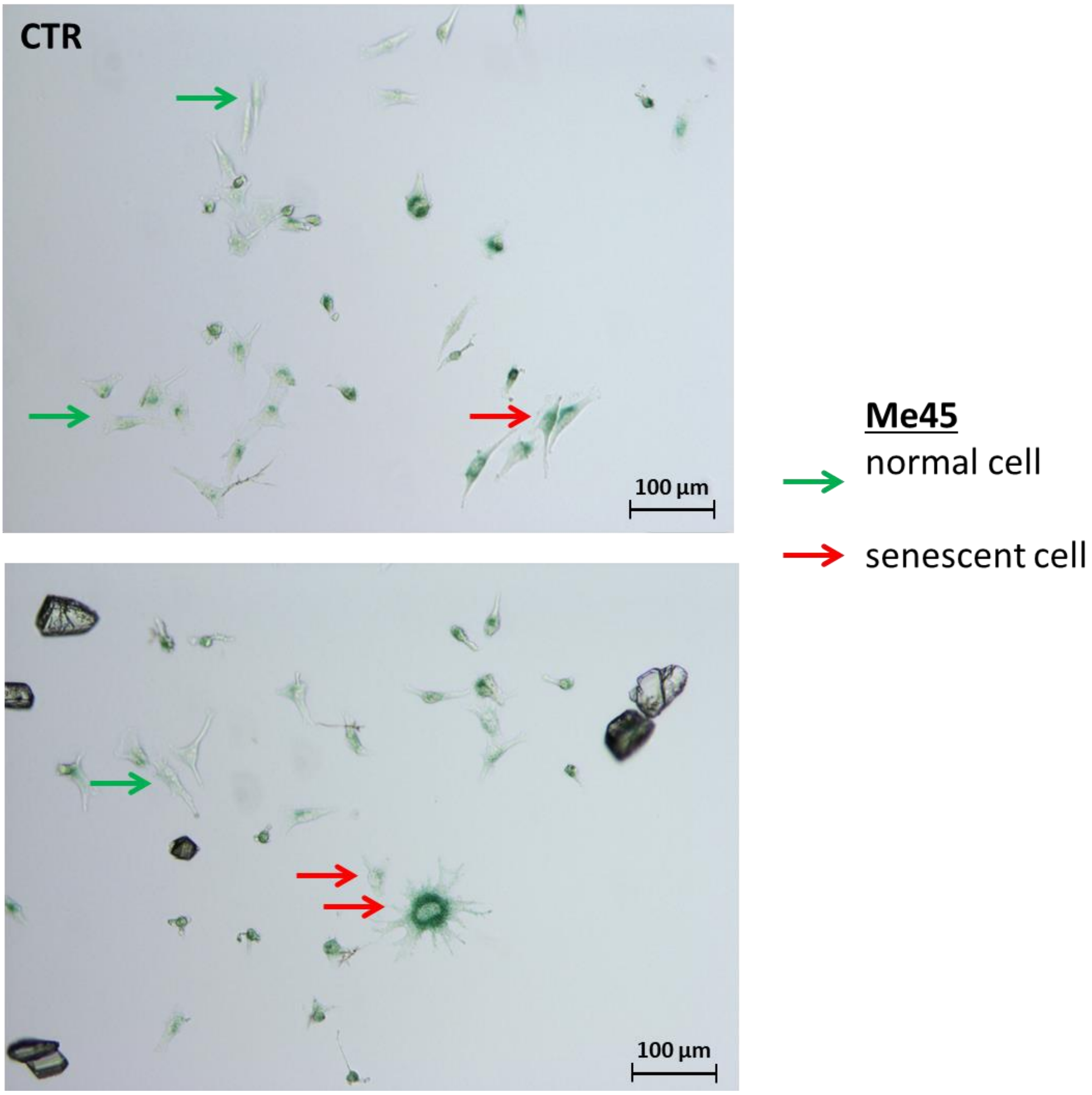

Figure S10. Me45 normal and senescent cells observed under the microscope after senescence test. Magnification 100 x, transit channel, scale bars 100 µm.
